# Pharmacokinetic-based failure of a detergent virucidal for SARS-COV-2 nasal infections

**DOI:** 10.21203/rs.3.rs-500168/v1

**Published:** 2021-05-14

**Authors:** Charles R. Esther, Kyle S. Kimura, Yu Mikami, Caitlin E. Edwards, Suman R. Das, Michael H. Freeman, Britton A. Strickland, Hunter M. Brown, Bronson C. Wessinger, Veerain C. Gupta, Kate Von Wahlde, Quanhu Sheng, Li Ching Huang, Daniel R. Bacon, Adam J. Kimple, Agathe S. Ceppe, Takafumi Kato, Raymond J. Pickles, Scott H. Randell, Ralph S. Baric, Justin H. Turner, Richard C. Boucher

**Affiliations:** University of North Carolina at Chapel Hill; Vanderbilt University Medical Center; University of North Carolina at Chapel Hill; University of North Carolina at Chapel Hill; Vanderbilt University Medical Center; Vanderbilt University Medical Center; Vanderbilt University Medical Center; Vanderbilt University Medical Center; Vanderbilt University Medical Center; Vanderbilt University Medical Center; Vanderbilt University Medical Center; Vanderbilt University Medical Center; Vanderbilt University Medical Center; University of North Carolina at Chapel Hill; University of North Carolina at Chapel Hill; University of North Carolina at Chapel Hill; University of North Carolina at Chapel Hill; University of North Carolina at Chapel Hill; University of North Carolina at Chapel Hill; University of North Carolina at Chapel Hill; Vanderbilt University Medical Center; University of North Carolina at Chapel Hill

**Keywords:** SARS-CoV-2, topical virucidal agent, irrigation

## Abstract

The nose is the portal for SARS-CoV-2 infection, suggesting the nose as a target for topical antiviral therapies. Because detergents are virucidal, Johnson and Johnson’s Baby Shampoo (J&J) was tested as a topical virucidal agent in SARS-CoV-2 infected subjects. Twice daily irrigation of J&J in hypertonic saline, hypertonic saline alone, or no intervention were compared (n = 24/group). Despite demonstrated safety and robust efficacy in *in vitro* virucidal assays, J&J irrigations had no impact on viral titers or symptom scores in treated subjects relative to controls. Similar findings were observed administering J&J to infected cultured human airway epithelia using protocols mimicking the clinical trial regimen. Additional studies of cultured human nasal epithelia demonstrated that lack of efficacy reflected pharmacokinetic failure, with the most virucidal J&J detergent components rapidly absorbed from nasal surfaces. This study emphasizes the need to assess the pharmacokinetic characteristics of virucidal agents on airway surfaces to guide clinical trials.

## Introduction

The nose is the primary portal for infection by SARS-CoV-2^1,2^. A fraction of inhaled SARS-CoV-2 impacts on nasal surfaces, which provide an ACE2 receptor-rich environment that promotes robust viral proliferation ^1^. From nasal surfaces, SARS-CoV-2 can spread locally to the olfactory region and more widely to the oral cavity and lung^1,3^. Accordingly, nasal protection represents an important strategy to limit SARS-CoV-2 infection and transmission^4^. In addition to physical means, *e.g.,* masks, topical administration of a spectrum of virucidal agents to nasal surfaces has been advocated for this purpose, including iodine, metal, and detergent-based molecular entities^5–7^.

Otolaryngologists utilize high-volume normal or hypertonic saline (HTS) rinses to cleanse the nasal cavity. A common agent added to saline rinses to provide gentle detergent activity is Johnson and Johnson’s (J&J) Baby Shampoo^8^. The J&J Baby Shampoo/saline (J&J/S) combination, typically 1/2 to 1 tsp/240 ml saline (8 oz), is safe and effective for treatment of chronic bacterial infections of the nasal cavity^9^. Because detergents are virucidal for enveloped viruses, including SARS-CoV-2, we tested J&J/S as a topical virucidal for the treatment of SARS-CoV-2 nasal cavity infections^5,10,11^.

## Results

The first component of this study investigated the *in vitro* safety and virucidal activity of clinically utilized dilutions of J&J/S^9^. Due to the urgency to limit SARS-CoV-2 nasal infections during the ongoing pandemic, a clinical trial of the safety and efficacy of J&J/S high-volume rinses in subjects with nasal SARS-CoV-2 cavity infections was then performed in parallel with the pharmacokinetic (PK) and virucidal pharmacodynamic (PD) studies of J&J/S in SARS-CoV-2-infected cultured human nasal epithelia (HNE).

**Figure 1:**
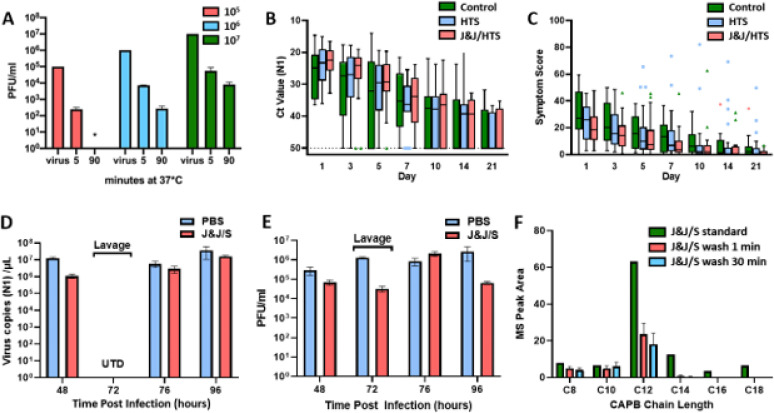
Preclinical, clinical, and pharmacokinetic data. Panel A depicts virucidal activity of J&J Shampoo (1/2 tsp J&J/240 ml saline) at a 1:1 dilution with SARS-CoV-2 viral stocks assayed varying viral titers. Initial SARS-CoV-2 stock titers ranged from 105–107 PFU/ml. *=p<0.05 vs. starting viral titer; ND=zero titer detected. Panel B depicts cross point (Ct) PCR-based measure of N1 primer-based viral load in the nasal cavity of SARS-CoV-2-infected subjects as a function of treatment group. N = 72; 24/group. Note, lower absolute Ct value reflects greater viral load. Panel C depicts nasal WURSS-21 symptom score of SARS-CoV-2-infected subjects as a function of treatment group over the study interval. Panel D depicts the nucleocapsid gene region 1 (N1) qPCR-based measure of SARS-CoV-2 viral copies in human nasal epithelial culture lavages collected at the designated times post D614G SARS-CoV-2 inoculation. Note, interference of the J&J Shampoo and the sample viral deactivation (8 M urea) protocol caused technical interference that obviated measurements of the 72 h pi J&J/S samples. Statistical analyses of changes for J&J/S and PBS lavage groups from 48 h pi values shown in Fig. S1H. Panel E depicts D614G SARS-CoV-2 viral titer data at the times post inoculum designated. Statistical analyses of changes in J&J/S and PBS 72 lavage groups compared to 48 h pi values shown in Fig. S1I. Panel F depicts results of mass spectroscopic analyses of application of J&J Shampoo (200 μl, 1/2 tsp/240 ml normal saline concentration) to cultured HNE and harvested 1 min and 30 min later for analysis. Cocamidopropyl betaine (CAPB) length denoted.

Safety studies revealed that exposure of HNE cultures to serial dilutions of J&J/S induced transient decreases in barrier function (transepithelial resistance) at concentrations above 1/2 tsp J&J/240 ml saline without changes in cell composition (Fig. S1A, B). Assays of virucidal activity demonstrated that non-toxic concentrations of J&J/S (1/2 tsp J&J/240 ml saline) were virucidal *in vitro* against NL63 coronavirus and respiratory syncytial virus (Fig. S1C, D). Notably, rapid and robust SARS-CoV-2 virucidal activity was observed when SARS-CoV-2 virus at varying titers was exposed to this concentration of J&J/S (Fig. 1A).

Having demonstrated *in vitro* safety and virucidal activity, an outpatient clinical trial of J&J/HTS nasal irrigation was performed in subjects with nasal swab qPCR-documented SARS-CoV-2 infection. Seventy-two subjects were randomized into three groups: 1) twice daily nasal irrigation with 1/2 tsp J&J Baby Shampoo in 240 ml HTS; 2) twice daily irrigation with HTS alone; or 3) no intervention (see Table 1, Fig. S1E). The primary study endpoint was qPCR-measured SARS-CoV-2 nasal viral load 4 h after irrigation over the three-week study interval. Other endpoints included patient-reported symptom scores assessed using a modified Wisconsin Upper Respiratory Symptom-21 Survey (WURSS-21) and daily temperatures.

No reductions in qPCR-measured nasal cavity viral load were observed in the intervention groups compared to control group (Fig. 1B, Fig. S1F). Similarly, no significant improvements in nasal symptoms were reported in either intervention group compared to the control group (Fig. 1C). No safety signals were observed, *e.g.,* no changes in WURSS-21 smell indices were observed in the irrigation groups to indicate irrigation-mediated spread of SARS-CoV-2 to olfactory epithelia (Fig. S1G), or daily temperature changes (not shown).

Insights into the disparity between the virucidal activity of J&J/S *in vitro* (Fig. 1A) and the absence of clinical effectiveness (Fig. 1B) emerged from the PK/PD studies of J&J/S in HNE cultures. For these studies, HNE cultures were inoculated at t=0 with D614G SARS-CoV-2 at an MOI of 0.1^12^. Samples for viral qPCR quantitation, utilizing the SARS-CoV-2 nucleoprotein (N1) primers employed in the clinical study, and viral titers were obtained via PBS lavage of HNE culture surfaces 48 h post inoculation (pi)^13^. At 72 h pi, J&J/S or PBS lavages were administered to HNE culture surfaces, fluids aspirated 10 min later, and titering performed. Note, viral qPCR was not technically accurate in virus-inactivated J&J Baby Shampoo lavage solutions. Importantly, the *in vitro* J&J/S lavage volume-to-HNE surface area ratio (200 μl/cm^2^) approximated the ratio *in vivo, i.e.,* 240 ml lavage spread over 150 cm^2^ nasal surface area^14^, and the 10 min *in vitro* J&J/S lavage residence time mimicked the *in vivo* contact time of lavages with nasal surfaces^8,15^. Viral qPCR and titering assays were performed on PBS lavages obtained 4 h after the 72 h J&J/S or PBS lavage, *i.e.,* at 76 h pi, and the next day at 96 h pi.

Like previous data^1^, PBS lavage samples revealed productive SARS-CoV-2 infection of HNE at 48 h pi (Fig. 1D, E), with little change in viral titer at 72 h pi (Fig. 1E, Fig. S1I). Surprisingly, the titers measured in the J&J/S lavage at 72 h pi were also not significantly different from those measured at 48 h in these samples (Fig. 1E, Fig. S1I, J). Furthermore, no differences in qPCR-measured viral load or viral titers from 48 h values were observed in the J&J/S or PBS lavaged HNE cultures over the 76–96 h pi interval (Fig. 1D, E, Fig. S1H, I).

Collectively, these data demonstrate that J&J/S exhibits virucidal activity against SARS-CoV-2 in a test tube *in vitro* (Fig. 1A) but not when administered onto nasal surfaces *in vitro* or *in vivo* (Fig. 1B, D, E). What is noteworthy about SARS-CoV-2-infected nasal surfaces is the continual production and release of SARS-CoV-2 virus onto nasal surfaces over time (Fig. 1E) ^1^. Accordingly, a virucidal agent must continually remain on nasal surfaces to produce durable and clinically meaningful virucidal activity in the nasal cavity. However, it is difficult to maintain effective concentrations of topical agents on nasal surfaces for two reasons. First, nasal surfaces are protected by a mucociliary transport system that clears nasal surfaces of topically deposited agents within 10 min^8,15,16^. Second, the nasal surfaces exhibit rapid transepithelial absorption of topically applied agents, a property accessed for drug delivery^17–19^.

We, therefore, studied the retention of topically applied J&J/S on nasal surfaces. J&J Baby Shampoo is a proprietary mix of agents. Mass spectrometric analysis of J&J/S indicated that the second listed agent after water, and dominant detergent, is cocamidopropyl betaine (CAPB) of varying chain lengths (Fig. S1K). Mass spectrometric analysis of PBS lavages obtained 1 min or 30 min after administration of J&J/S to HNE surfaces revealed that the longer chain CAPB components were absorbed from HNE surfaces within 1 min of administration (Fig. 1F). These data suggest that the failure of J&J/S to reduce SARS-CoV-2 titer in the HNE studies (Fig. 1D, E) reflected the rapid transnasal absorption of the longer chain, more active virucidal components of J&J/S from the lavage fluid (Fig. S1L)^11,20^.

## Discussion

Our data do not support use of J&J/S as a topical virucidal agent for treatment of active SARS-CoV-2 nasal infections. The failure of J&J/S to treat SARS-CoV-2 did not reflect a defect in the intrinsic virucidal activity of this agent. Rather, the result reflected a PK failure to maintain for prolonged intervals the concentrations of J&J virucidal ingredients on nasal surfaces required to inactivate SARS-CoV-2 virions continuously being shed onto nasal surfaces. SARS-CoV-2-infected nasal surfaces continually produce and release SARS-CoV-2 virus onto nasal surfaces over time (Fig. 1E)^1^. Accordingly, a virucidal agent must continually remain on nasal surfaces to produce durable and clinically meaningful virucidal activity in the nasal cavity. However, it is difficult to maintain effective concentrations of topical agents on nasal surfaces for two reasons. First, nasal surfaces are protected by a mucociliary transport system that clears nasal surfaces of topically deposited agents within 10 min^8,15,16^. Second, the nasal surfaces exhibit rapid transepithelial absorption of topically applied agents, a property accessed for drug delivery^17–19^.

These data suggest that the failure of J&J/S to reduce SARS-CoV-2 titer in the HNE studies reflected primarily the rapid transnasal absorption of the longer chain, more active virucidal components of J&J/S from the lavage fluid. Longer chain detergents have greater antiviral activity but also readily absorb onto cell surfaces^11,20^, which we show limits their potential as antiviral agents. Although our studies focused on CAPB as the dominant detergent in J&J, similar findings likely apply to similar detergents in J&J or other products. We also cannot rule out the possibility that mucociliary transport may have also contributed to the lack of efficacy in the clinical trial.

This study emphasizes the need to assess *a priori the* PK characteristics of virucidal agents considered for topical use against SARS-CoV-2 nasal cavity infections. Cell culture systems such as the ones utilized in our study can be utilized to rapidly evaluation topical antiviral agents to determine which retain their efficacy when applied to human airway epithelia, particularly when exposure times are limited to mimic the impact of mucociliary clearance. Such studies can be utilized to inform clinical trials for SARS-CoV-2 and other respiratory viral infections.

## Methods

Methods are described briefly below, with further information in the supplement.

### *In Vitro* Safety, Pharmacodynamic, and Pharmacokinetic Studies

#### Safety Studies in Airway Epithelia:

Apical surfaces of well-differentiated air–liquid interface human nasal epithelial (HNE) cultures were exposed to 5 μL of various dilutions of J&J/S for 5 min, washed with 600 μL of cell culture media^1^, and transepithelial resistance (R_t_) measured utilizing an EVOM (World Precision Instruments). Parallel cultures after J&J/S exposure were fixed in 4% PFA and processed for whole mount histologic analyses ^1^.

#### Virucidal Assays in Vero and Cultured Human Nasal Cells:

SARS-CoV-2 D614G virus stocks at 10^7^ PFU/ml were incubated with J&J at 1:1 or 1:100 dilutions for 90 min at 37°C, then serially diluted in PBS for plaque assay on Vero cells^1^. Virucidal activity of J&J Baby Shampoo was also assessed in recombinant NL63-coronavirus (NL63-CoV) ^21^ and Respiratory Syncytial Virus (RSV)^22^ expressing GFP. 100 ml of NL63 or RSV were mixed with PBS or J&J/S, incubated at 37°C for 5 min or 90 min, then virus titers determined by Median Tissue Culture Infectious Dose (TCID50/ml) on Vero cells.

Well differentiated HNE cultures were inoculated with 200 μl of SARS-CoV-2 D614G at an MOI of 0.1, incubated at 37°C for 90 min, following which the inoculum was removed and cultures lavaged 2X with 500 μl PBS to remove residual virus. Apical lavages were performed with 200 μl PBS for 10 min at 37°C at 48 hpi, 76 hpi, and 96 hpi as described in the text. HNE were also lavaged with a J&J/S or PBS at 72 hpi. Apical lavages were stored at −80°C until analyzed by qPCR to measure viral load and plaque assay to determine viral titer.

Individual surfactant components of CAPB including octanoylamide propylbetaine (C8 chain length), lauroylamide propylbetaine (C12 chain length), and palmitoylamide propylbetaine (C18 chain length) were obtained from Toronto Research Chemicals (Toronto, CA). SARS-CoV-2 D614G virus stock at 10^7^ PFU/ml was incubated with each surfactant individually at 1 mg/mL, 0.1 mg/mL, or 0.01 mg/mL for 90 min at 37°C. Titers of active virus post treatment were determined by the plaque titer assay as described above.

##### qPCR Quantitation of SARS-CoV-2 Viral Load from HNE Cultures:

Apical culture lavages were inactivated with urea, and RNA was isolated using Direct-zol RNA Kits (#R2073, ZYMO RESEARCH). Briefly, 100 μL apical lavage was mixed with 300 μL TRI reagent followed by 400 μL 99% ethanol, transferred to the spin column, centrifuged with wash buffer then eluted with 50 μL RNase-free water. Total RNA was reversed transcribed into cDNA with iScript Reverse Transcription Supermix for RT-qPCR (#1708841, Bio-Rad, CA, USA) and virus copy number was quantitated using the SARS-CoV-2 (2019-nCoV) CDC qPCR Probe Assay kit (#10006770, Integrated DNA Technologies).

##### LCMS/MS:

Five μL of a 1:100 dilution of J&J Baby Shampoo was analyzed using chromatographic and mass spectrometric conditions as previously described^23^. Full scans were run in positive mode with electrospray interface (ESI).

##### Statistical Analyses of in vitro studies:

For transepithelial airway culture resistance, the Dunnet test (control = PBS) was utilized to assess differences in R_t_ as a function of J&J/S concentrations at serial time points. To analyze the effects of J&J/S vs. PBS on magnitudes of SARS-CoV-2 infection of HNE, the changes from 48 h at each successive time point were analyzed with a repeated measure model. Interaction of time and treatment (J&J/S vs PBS) was explored and 48 h values were included as covariates. Post-hoc comparisons of treatments at each time point were adjusted with Holm. P values < 0.05 were considered significant. Changes in viral titers with J&J or CAPB lipids were assessed using one sample T-tests against expected titers, with Bonferroni corrections to account for multiple testing.

### Clinical Trial of J&J Baby Shampoo

#### Study Population and Enrollment:

The clinical study was approved by the Vanderbilt University Medical Center Institutional Review Board and Biosafety Committee and registered on clinicaltrials.gov (NCT04347538). A CONSORT diagram for the study is shown in Fig. S1E. Patients with a positive qualitative qRT-PCR test for the SARS-CoV-2 virus were randomized to one of three treatment groups: 1) no intervention, 2) hypertonic nasal saline irrigations BID, and 3) hypertonic nasal saline irrigations with 1/2 teaspoon surfactant (Johnson’s Baby Shampoo; Johnson & Johnson Inc.; New Brunswick, NJ) BID. Hypertonic saline solution consisted of 240 mL of distilled water with 2 packets of NeilMed brand buffered salt (NeilMed Pharmaceuticals; Santa Rosa, CA). Nasal lavage was performed in each nostril using NeilMed brand Sinus Rinse bottles. Randomization, enrollment, and registration took place via REDCap (Vanderbilt University, Nashville TN). Further data, including statistical analyses, are in the supplement.

## Supplementary Material

Supplement 1

## Figures and Tables

**Table 1.** Clinical and demographic characteristics of study participants.

	No Intervention (n=24)	Hypertonic Saline (n=24)	Saline + Surfactant (n=24)	p value

**Age**	39 ± 15	39 ± 15	44 ± 18	0.68
	
**Sex, no. male (%)**	14 (58)	12 (50)	9 (38)	0.35

**BMI**	29.6 ± 7.2	28.5 ± 6.3	28.9 ± 5.9	0.89

**Symptomatic days before diagnosis**	2.0 (1.0–3.0)	2.5 (1.0–4.8)	2.0 (1.0–3.5)	0.79

**Smoking, no. (%)**	1 (4)	2 (8)	2 (8)	0.81

**Comorbidities**				

**Diabetes, no. (%)**	3 (12)	0 (0)	3 (12)	0.2

**Heart disease, no. (%)**	2 (8)	0 (0)	1 (4)	0.18

**Hypertension, no. (%)**	4 (17)	4 (17)	6 (25)	0.7

**Chronic lung disease, no. (%)**	4 (17)	3 (12)	2 (8)	0.68
