# Supplementary material for: Pharmacokinetic-based failure of a detergent virucidal for SARS-COV-2 nasal infections
Source: Res Sq. 2021 May 14:rs.3.rs-500168. Preprint. [Version 1] doi: 10.21203/rs.3.rs-500168/v1 (PMC8132247; doi:10.21203/rs.3.rs-500168/v1)
Supplement: Supplement 1 [file 11d2d28d955f57eec02dd65a.docx]

**Pharmacokinetic-Based Failure of a Detergent Virucidal for SARS-CoV-2 Nasal Infections**

**Supplemental Data**

**Methods**

***In Vitro* Safety, Pharmacodynamic (Virucidal), and Pharmacokinetic Studies**

***Safety Studies in Airway Epithelia:*** The apical surfaces of well-differentiated air–liquid interface human nasal epithelial (HNE) cultures were exposed to 5 μL of three different dilutions of J&J Baby Shampoo (0.5 ml J&J/240 ml saline; 2.5 ml J&J/240 ml saline; or 5 ml J&J/240 ml saline) for 5 min and subsequently washed with 600 μL of cell culture media^1^. Note, 1 tsp = 5 ml. Transepithelial resistance (R_t_) was measured at baseline, 5 min, 1 h, 2 h, and 24 h after PBS or J&J Baby Shampoo administration utilizing an EVOM (World Precision Instruments). Parallel cultures after J&J/S exposure were fixed in 4% PFA and processed for whole mount histologic analyses of HNE cell composition as previously described^1^.

***Virucidal Assays in Vero Cells and Cultured Human Nasal Epithelial Cells:*** To determine the virucidal activity of a clinical dose J&J shampoo with respect to SARS-CoV-2 D614G, virus stocks at a starting concentration of 10^7^ PFU/ml were incubated with J&J at 1:1 or 1:100 dilutions for 90 min at 37ºC. Following the 90 min incubation, the virus + shampoo mixtures were immediately serially diluted 1:10 in PBS for a 6-fold dilution plaque assay on Vero cells^1^. After 3 d, plates were stained with 1X neutral red and plaques counted.

To test the virucidal activity of J&J Baby Shampoo on other enveloped respiratory viruses of relevance to virus infection of the nasal epithelium, previously described recombinant NL63-coronavirus (NL63-CoV)^2^ and Respiratory Syncytial Virus (RSV)^3^ expressing GFP were studied. Viral stocks of NL63-CoV and RSV were generated in LLC-MK2 cells and HEp-2 cells, respectively and the Median Tissue Culture Infectious Dose (TCID50/ml) determined on Vero cells. NL63-CoV and RSV titers were Log10 TCID50/ml of 8.0 and 6.0, respectively. To determine the virucidal activity of J&J Baby Shampoo on these viruses, 100 μl of NL63 or RSV were mixed with either 100 μl of PBS or Shampoo (1 tsp J&J/250 ml PBS) or 10 ml of PBS/Shampoo (1/2 tsp J&J/250 ml PBS) and incubated at 37ºC for 5 min or 90 min after which the virus titers in these solutions were determined by TCID50 on Vero cells in 96-well plates.

The *in vitro* effect of a clinical dose J&J wash on HNE cultures was determined in parallel with *in vivo* clinical lavage studies. HNE cultures were propagated in PneumoCult media until mature and fully ciliated^1^. HNE cultures were inoculated with 200 µl of SARS-CoV-2 D614G at an MOI of 0.1. Cultures were incubated at 37ºC for 90 min, following which, the inoculum was removed and cultures lavaged 2X with 500 µl PBS to remove residual virus. At 48 h post infection (hpi), each culture was lavaged with 200 µl PBS for 10 min at 37ºC (all apical lavages were performed in this manner). Apical lavages were collected and stored at -80ºC. At 72 hpi, cultures were split into two groups: half receiving an apical lavage of a clinical dose J&J shampoo; half receiving a PBS lavage. Four hours following these lavages, cultures were lavaged a second time with 200 µl PBS. Culture was continued until 96 hpi when final PBS apical lavages were performed. Apical lavages were then subjected to qPCR measurement of viral load and titered by plaque assay to determine viral titer.

To determine the virucidal activity of individual surfactant components of CAPB of various chain lengths, octanoylamide propylbetaine (C8 chain length), lauroylamide propylbetaine (C12 chain length), and palmitoylamide propylbetaine (C18 chain length) were obtained from Toronto Research Chemicals (Toronto, CA). SARS-CoV-2 D614G virus stock at a starting concentration of 10^7^ PFU/ml was incubated each surfactant individually at concentrations of 1 mg/mL, 0.1 mg/mL, or 0.01 mg/mL for 90 min at 37ºC. Titers of active virus post treatment were determined by the plaque titer assay as described above.

***qPCR Quantitation of SARS-CoV-2 Viral Load from HNE Cultures:*** Apical culture lavages were mixed with 16 M urea at a 1:1 ratio (final concentration 8 M urea) to inactivate the virus. The RNA isolation from the apical lavage was performed using Direct-zol RNA Kits (#R2073, ZYMO RESEARCH) following the manufacture’s instructions. Briefly, 100 μL of the apical lavage was mixed with 300 μL of TRI reagent followed by 400 μL of 99% ethanol. The mixture was transferred to the spin column and centrifuged with the wash buffer. The RNA was eluted with 50 μL of RNase-free water. Total RNA was reversed transcribed into cDNA with iScript Reverse Transcription Supermix for RT-qPCR (#1708841, Bio-Rad, CA, USA) according to the manufacturer’s instructions. All samples were reverse transcribed from the same volume of RNA template. The virus copy number in the samples was quantitated using the SARS-CoV-2 (2019-nCoV) CDC qPCR Probe Assay kit (#10006770, Integrated DNA Technologies) following the manufacturer’s instructions.

***LC-MS/MS:*** Five µL of a 1:100 dilution of J&J Baby Shampoo was analyzed using chromatographic and mass spectrometric conditions as previously described [doi: 10.1152/ajplung.00398.2016]. Full scans were run in positive mode with electrospray interface (ESI).

***Statistical Analyses of in vitro studies:*** For transepithelial airway culture resistance, the Dunnet test (control = PBS) was utilized to assess differences in R_t_ as a function of J&J/S concentrations at serial time points.

To analyze the effects of J&J/S vs. PBS on magnitudes of SARS-CoV-2 infection of cultured human nasal epithelia, the changes from 48 h at each successive time point were analyzed with a repeated measure model. Interaction of time and treatment (J&J/S vs PBS) was explored and 48 h values were included as covariates. Post-hoc comparisons of treatments at each time point were adjusted with Holm. P values < 0.05 were considered significant. Changes in viral titers with J&J or CAPB lipids were assessed using one sample T-tests against expected titers, with Bonferroni corrections to account for multiple testing.

**Clinical Trial of J&J Baby Shampoo**

***Study Population and Enrollment:*** The clinical study was approved by the Vanderbilt University Medical Center Institutional Review Board and Biosafety Committee and registered on clinicaltrials.gov (NCT 04347538). A CONSORT diagram for the study is shown in Fig. S1E and demographics of study population shown in Table S1. Inclusion criteria included a positive qualitative qRT-PCR test for the SARS-CoV-2 virus at Vanderbilt University Medical Center (VUMC) or affiliated testing centers, age of 18 years or greater, and planned self-quarantine after infection within a 30-mile radius of VUMC. Exclusion criteria included inpatient admission, current use of nasal saline irrigations or other intranasal medications, and inability to perform saline irrigations/nasal swabs in separate bathroom away from household contacts. Any patient requiring hospitalization during the course of the study terminated data collection concomitant with hospitalization. Enrollment was performed via telephone on a rolling basis, predicated on a positive qRT-PCR test in the preceding 24 hours. Patients were randomized to one of three treatment groups: 1) no intervention, 2) hypertonic nasal saline irrigations BID, and 3) hypertonic nasal saline irrigations with ½ teaspoon (2.5 mL) surfactant (Johnson’s Baby Shampoo; Johnson & Johnson Inc.; New Brunswick, NJ) BID. Hypertonic saline solution consisted of 240 mL of distilled water with 2 packets of NeilMed brand buffered salt (NeilMed Pharmaceuticals; Santa Rosa, CA). Nasal lavage was performed in each nostril using NeilMed brand Sinus Rinse bottles. Randomization, enrollment, and registration took place via REDCap (Vanderbilt University, Nashville TN).

***Study Protocol:*** Patients were trained in self-performance of mid-turbinate binaral swab collection using written directions and a standardized web application. A total of seven sterile individually wrapped nasal swabs (FLOQSwabs; Copan Diagnostics; Murrieta, CA) were delivered with single use collection and preservation vials (OMR 110; DNA Genotek Inc; Ottawa, ON, Canada) All materials were provided to participants within 24 hours of enrollment. Patients performed swab collection on days 1, 3, 5, 7, 9, 14, and 21. Swabs were performed at least 4 hours after saline rinses. Patients also recorded their daily temperature (same time each day) and completed a symptom questionnaire based on the validated Wisconsin Upper Respiratory Symptom Survey 21 (WURSS-21)^4^ (Fig. S2). The survey was modified to capture symptoms prevalent during SARS-CoV-2 infection that may be less common during other respiratory virus infections. Added symptoms included eye redness/pain, headache, sputum production, coughing blood, shortness of breath, nausea/vomiting, muscle/joint pain, chills, and alteration of smell/taste. Each survey question is scored using a modified Likert scale with 0 indicating a lack of symptoms and 7 indicating severe symptoms. At the completion of the study, nasal swabs and symptom questionnaires were collected from participants at their residences. SARS-CoV-2 quantitative qRT-PCR testing was performed as previously described^5^. Both the SARS-CoV-2 nucleocapsid gene region 1 (N1) and nucleocapside gene region 2 (N2) were amplified for detection. RNA quality and quantity were examined using RNase P.

***Studies of Nasal Swab Viral Load:*** SARS-CoV-2 viral load was measured in mid-turbinate nasal swabs collected 4 h after lavage on d 1, 3, 5, 7, 10, 14, and 21 post-study initiation using validated primers widely used for diagnostic testing (N1, N2, RP)^5^. No significant differences in Ct values were identified at any time point for either the N1 or N2 primer.

***Power Calculations and Statistical Analyses of the Clinical Trial:*** Power analyses utilized data from studies of nasal SARS CoV-2 loads over time in untreated individuals^6,7^ and studies of nasal viral load of non-SARS CoV-2 coronoviruses with saline irrigation treatment^8^ to estimate mean treatment effects of 1.51 and 2.23 for control^2^ and treatment, respectively (units for viral load measured in log CT PCR) with a standard deviation of 0.97. Using an alpha of 0.025 (Bonferroni correction for testing intervention 1 vs. control and intervention 2 vs. control, overall alpha = 0.05), a minimum sample size of 36 subjects was found to be necessary to generate a power of 0.80.

Participants’ demographic, baseline characteristics and outcomes measures were summarized with median and interquartile range for continuous variables, or frequency and percentage for categorical variables. Differences among the three groups were assessed using the Kruskal-Wallis test for continuous variables and Pearson Chi-squared test for categorical variables. The primary outcome was qRT-PCR-measured nasal SARS-CoV-2 viral load. The secondary outcomes included patient-reported symptom score, daily temperature, and viral shedding, with anosmia included as a safety signal. For calculating the viral shedding value, CT values were capped at 40 and were converted to log10 values (change in values from CT at day 1 to the maximum value of CT / days between two values).  A CT value greater than 50 was considered undetectable. To account for the large number of undetectable values at later time points a multivariable longitudinal regression model with parametric survival was used for the primary outcome. Generalized estimating equations (GEE) were used to account for the correlation among records collected from the same participant. Potential confounders included day, RNAse P, age, gender, BMI and symptom score at day 1, and were adjusted in the model. To allow nonlinear associations with the outcome and day, the restricted cubic splines with 3 knots was used for day.  Missing values for regression model covariates were imputed using multiple imputation using chained equations (MICE). Two-sided P-values less than or equal to .05 were considered statistically significant. Statistical analyses were performed using R version 4.0 in addition to rms, Hmisc and survival packages (R Foundation for Statistical Computing).

We performed a planned interim analysis after the first 45 patients completed the study protocol. The trial interventions were deemed of minimal risk by the Vanderbilt Institutional Review Board and as such did not necessitate external data safety monitoring. We, therefore, did not establish a pre-specified stopping rule based on adverse events, and instead the study investigators reviewed enrollment data every 2 weeks to monitor for any adverse events and drop-outs. The interim analysis permitted validation of adequate genetic material in the self-swab samples and also allowed for an analysis for futility based on the primary (change in viral load from day 1 to day 21) and secondary (time to symptom resolution) outcomes via calculation of probability of superiority and conditional power. Following interim analysis, further enrollment was discontinued based on these criteria and final data analysis was performed after all currently enrolled patients had completed the study.

**Supplementary Figure**


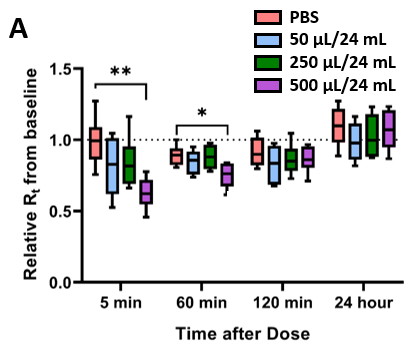

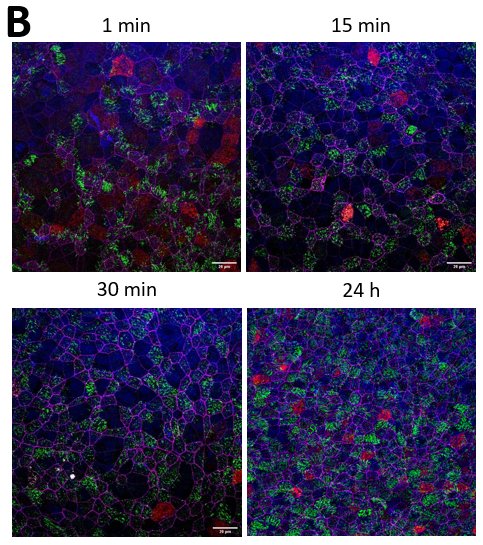


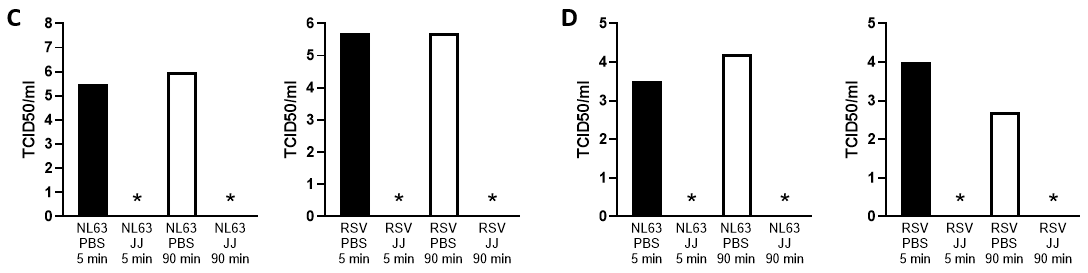


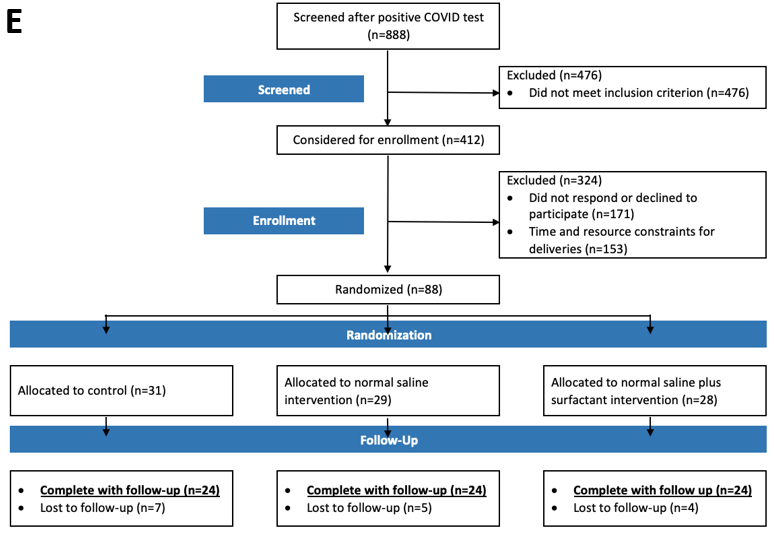


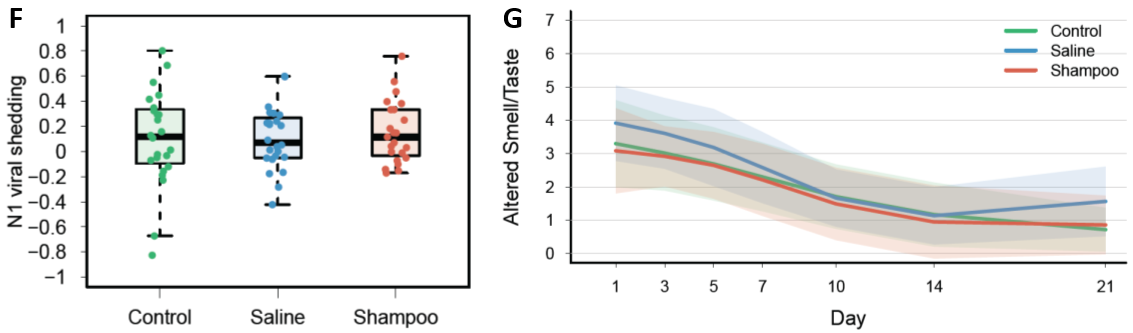


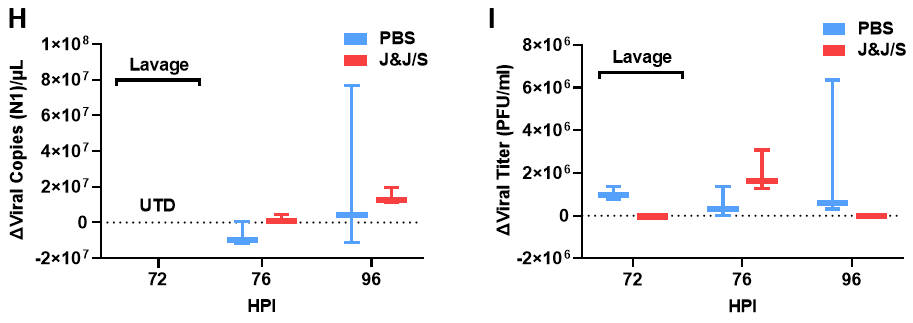


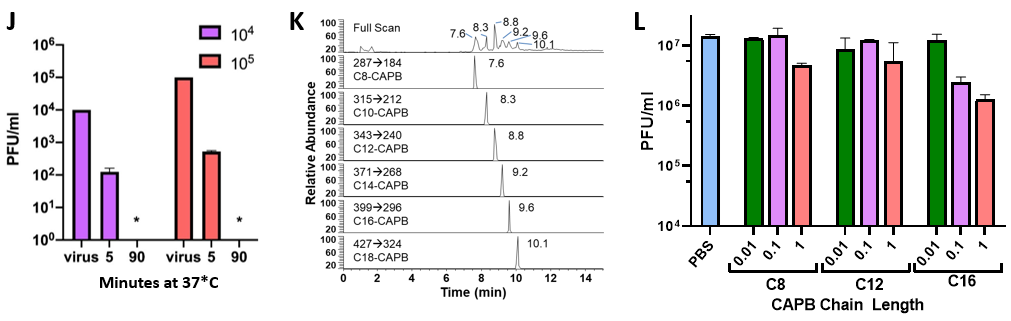


**Figure S1. Panel A** depicts the transepithelial resistance (R_t_) relative to baseline after administration of J&J Shampoo/normal saline at three concentrations (0.1, 0.5, or 1 tsp in 240 ml of normal saline) or phosphate-buffered saline (PBS) to cultured human nasal epithelial (HNE) cells. * depicts p<0.05 different than baseline, ** depicts p<0.01. **Panel B** depicts whole-mount immunofluorescent staining images of human bronchial epithelial (HBE) cultures. Representative fluorescent images of HBE cells after the administration of J&J Baby Shampoo at a concentration of 1/2 tsp/240 ml of phosphate-buffered saline (PBS) for 1 min, 15 min, 30 min, or 24 h shown. The HBE cells were fixed and stained with α-Tubulin (cilia; green), CCSP (club cell; red), Phalloidin (f-actin; magenta), MUC5B (secretory cell; white), and DAPI (nuclei; blue). Scale bar: 20 μm. **Panels C/D** depict the viral titers of NL63 coronavirus (NL63, **C**) and Respiratory Syncytial Virus (RSV, **D**) determined as the Median Tissue Culture Infectious Dose (TCID50/ml) after virus aliquots were exposed to PBS (control) or J&J Baby Shampoo for 5 min or 90 min at 1:1 volume dilution (*left panels*) or 1:100 volume dilution (*right panels*) (both at final concentration of 1/2 tsp/240 ml shampoo). * denotes values below the limit of detection (Log10TCID50/ml of 1.5). **Panel E** depicts a consort diagram describing the screened and enrolled subjects for the randomized study of J&J/S, HTS, and no-intervention control groups. Diagram is based on the CONSORT transparent reporting of trials (<http://www.consort-statement.org/>). **Panel F** depicts time-dependent change in viral shedding for each of the three treatment groups, defined as log 10 (change in CT value between day 1 and maximum (T value) day between the two values. N = 24/treatment group. **Panel G** depicts the Wisconsin Upper Respiratory Symptom-21 survey data describing altered smell/taste in the SARS-CoV-2-infected subjects for each of the three treatment groups over the study interval. N = 24/group. **Panel H** depicts the change from 48 h pi in the SARS-CoV-2 nucleocapsid gene region 1 (N1) qPCR-measured viral load at 76 and 96 h pi for groups lavaged at 72 h pi with PBS or J&J/S. UTD = unable to determine. **Panel I** depicts the change in human nasal epithelial (HNE) cell viral titers from the 48 h pi value at 72, 76, and 96 h post-inoculation with DG14G SARS-CoV-2 virus at an MOI = 0.1. Note, at 72 h the lavage solution was either J&J/S (1/2 tsp/240 ml saline) or phosphate-buffered saline (PBS). **Panel J** depicts SARS-CoV-2 viral titers measured after exposure of SARS-CoV-2 viral stocks (10^6^ or 10^7^ PFU/ml initial) diluted 1:100 into a 1/2 tsp J&J/240 ml normal saline solution. Note, the virucidal activity of detergents is a function of the mass of available detergent/target virions. Thus, at the virus (~ 2 µl volume on HNE surfaces) to lavage ratio (200 µl), *i.e.*, a 1:100 dilution, utilized in the HNE protocols, J&J/S should be equally or more effective than at the 1:1 dilution used in our routine titering assay (Fig. 1B). However, to verify this assumption, J&J/S titering for virucidal activity was also performed at a 1:100 dilutions, *i.e.*, a 1:100 dilution of SARS-CoV-2 stock virus into J&J/S and tittered on Vero cells. The data presented in this figure confirmed the virucidal efficacy of J&J/NS at 1:100 dilution. *=p<0.05 vs. starting viral titer; ND=zero titer detected. **Panel K** depicts mass spectrographic analyses of detergent composition of Johnson and Johnson’s Baby Shampoo. **Panel L** depicts the relative virucidal activities of three detergents contained with J&J Baby Shampoo that vary by chain length. *=p<0.05 vs. starting viral titer (PBS).


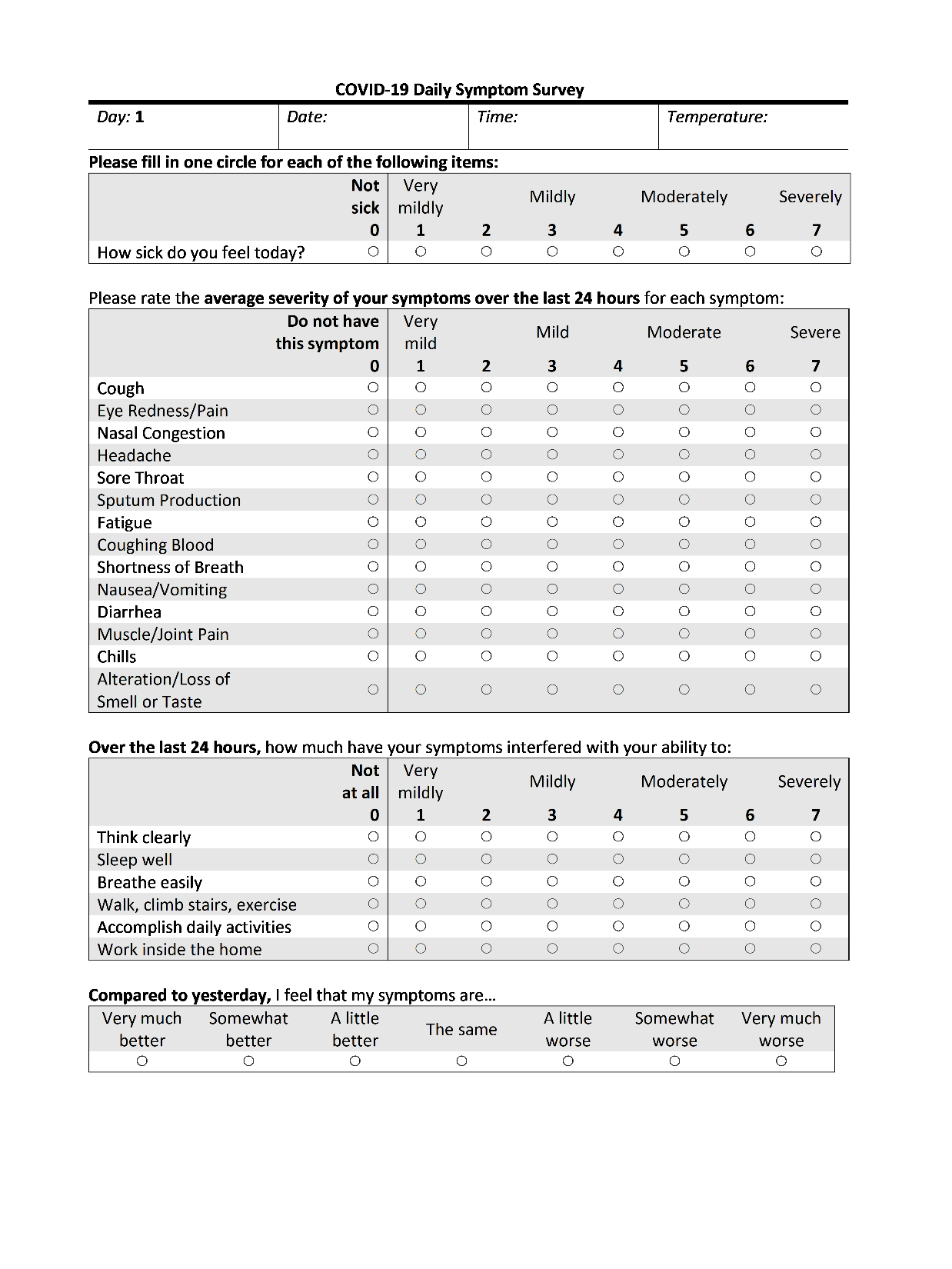


**Figure S2.** COVID-19 daily symptom survey (modified from Wisconsin Upper Respiratory Symptom Survey – 21^8^).

**References**

1. Hou, Y.J.*, et al.* SARS-CoV-2 Reverse Genetics Reveals a Variable Infection Gradient in the Respiratory Tract. *Cell* **182**, 429-446 e414 (2020).

2. Donaldson, E.F.*, et al.* Systematic assembly of a full-length infectious clone of human coronavirus NL63. *J Virol* **82**, 11948-11957 (2008).

3. Zhang, L., Peeples, M.E., Boucher, R.C., Collins, P.L. & Pickles, R.J. Respiratory syncytial virus infection of human airway epithelial cells is polarized, specific to ciliated cells, and without obvious cytopathology. *J Virol* **76**, 5654-5666 (2002).

4. Barrett, B.*, et al.* The Wisconsin Upper Respiratory Symptom Survey is responsive, reliable, and valid. *Journal of clinical epidemiology* **58**, 609-617 (2005).

5. Rosas-Salazar, C.*, et al.* SARS-CoV-2 Infection and Viral Load are Associated with the Upper Respiratory Tract Microbiome. *The Journal of allergy and clinical immunology* (2021).

6. Zheng, S.*, et al.* Viral load dynamics and disease severity in patients infected with SARS-CoV-2 in Zhejiang province, China, January-March 2020: retrospective cohort study. *BMJ* **369**, m1443 (2020).

7. Yu, X.*, et al.* SARS-CoV-2 viral load in sputum correlates with risk of COVID-19 progression. *Crit Care* **24**, 170 (2020).

8. Ramalingam, S., Graham, C., Dove, J., Morrice, L. & Sheikh, A. A pilot, open labelled, randomised controlled trial of hypertonic saline nasal irrigation and gargling for the common cold. *Scientific reports* **9**, 1015 (2019).
